# RETRACTION: *Ex Vivo* Stromal Cell-Derived Factor 1-Mediated Differentiation of Mouse Bone Marrow Mesenchymal Stem Cells into Hepatocytes Is Enhanced by Chinese Medicine Yiguanjian Drug-Containing Serum

**DOI:** 10.1155/ecam/9812485

**Published:** 2025-04-24

**Authors:** Evidence-Based Complementary and Alternative Medicine

RETRACTION: L. Fu, B. Pang, Y. Zhu, L. Wang, A. Leng, H. Chen, “*Ex Vivo* Stromal Cell-Derived Factor 1-Mediated Differentiation of Mouse Bone Marrow Mesenchymal Stem Cells into Hepatocytes Is Enhanced by Chinese Medicine Yiguanjian Drug-Containing Serum”, Evidence-Based Complementary and Alternative Medicine, 2016, https://doi.org/10.1155/2016/7380439.

The above article, published online on 14 April 2016 in Wiley Online Library (https://wileyonlinelibrary.com), has been retracted by agreement between the handling Editor, Yuewen Gong, and John Wiley & Sons Ltd. The retraction has been agreed due to concerns originally raised on PubPeer [1], where the article was found to have concerns with the figures. Specifically:

• Figure 4(b) (panel a4) appears to have a duplicated region with Figure 6(c) (panel b3)
• Figure 1(e) appears to be duplicated in Figure 3(a)

The authors stated that this error was due to carelessness in the preparation of the manuscript and apologized for not identifying it. A replacement image for Figure 6(c) was provided; however, the editorial board have outstanding concerns regarding the reliability of the data. The article is therefore being retracted due to the above concerns with the agreement of the editorial board.

The authors do not agree to the retraction.
